# The complete mitochondrial genome of the pyrophilous jewel beetle *Melanophila acuminata* (Coleoptera: Buprestidae)

**DOI:** 10.1080/23802359.2021.1899079

**Published:** 2021-03-19

**Authors:** Xujian Peng, Jing Liu, Zheng Wang, Qingbin Zhan

**Affiliations:** a Department of Public Order Policing Science, Nanjing Forest Police College, Nanjing, China; b Faculty of Humanities and Social Sciences, Nanjing Forestry University, Nanjing, China; c Department of Information Science and Technology, Nanjing Forest Police College, Nanjing, China;; d Department of Criminal Science and Technology, Nanjing Forest Police College, Nanjing, China;; e Key Laboratory of Wildlife Evidence Technology, State Forest and Grassland Administration, Nanjing, China

**Keywords:** *Melanophila*, fire-loving, pyrophilous insect, mitochondrial genome

## Abstract

The complete mitochondrial of genome *Melanophila acuminata* (DeGeer 1774) is a typical double-stranded circular molecule of 15,853 bp (GenBank accession number: MW287594). All tRNA genes, ranging from 62 to 72 bp, can be folded into typical clover-leaf secondary structure except for *tRNA^Ser(AGN)^*. The control region is 1,080 bp long with an A+T content of 87.5%. The phylogeny tree is monophyletic among 19 related species. The *Melanophila acuminata* cluster was more closely related to *Chrysochroa fulgidissima*. This mitochondrial genome can be used for further analyses of Buprestidae mitochondrial comparative genomics to improve the understanding of diverse coleopteran species.

Fire-loving (pyrophilous) insects depend on forest fires for their reproduction. They were mainly found in freshly burnt habitats. *Melanophila acuminata* is one of the most famous pyrophilous insects (Evans 1964). It was equipped with a thoracic IR receptor. A large number of *M. acuminata* have been attracted by a large smelter plant where the next coniferous forest was about 50 miles away (Schmitz and Bousack 2012). In this study, the specimens of *M. acuminata* were collected from Chengde, Hebei Province (41°4′22.3′′N 117°55′30.4′′W) in April 2018. All specimens and isolated DNA samples were deposited at the key laboratory of wildlife evidence technology state forest and grassland administration (specimen code NFPC 20190514). Total genomic DNA was extracted from the muscle of the specimen with the QIAamp DNA Mini Kit (QIAGEN, Hilden, Germany) following the manufacturer’s instruction. The whole genomic DNA was sequenced by Illumina HiSeq 6000 platform (Illumina, San Diego, CA). The complete mitogenome was annotated using the MITOS web server (Bernt et al. 2013).

The complete mitochondrial genome of *M. acuminata* (DeGeer 1774) was sequenced in this study. The complete mitochondrial genome is a typical double-stranded circular molecule of 15,853 bp (GenBank accession number: MW287594). It includes the entire set of 37 genes (i.e. 13 protein-coding genes, 22 tRNA genes, and 2 rRNA genes) usually present in animal mitochondrial genomes and a control region. Gene order is identical to that of the putative ancestral arrangement of insects and other coleopterans (Boore 1999; Li et al. 2012; Cameron 2014; Duan et al. 2017). There are a total of 149 overlapped nucleotides between genes in 17 locations, ranging from 1 to 62 bp in length; while there are in total 143 bp intergenic nucleotides in 13 locations, ranging from 1 to 21 bp in length.

ATN, GTG, TTG, and GTT are accepted canonical mitochondrial start codons for invertebrate mtDNAs, and most of PCGs exhibit these start codons (Wolstrnholme 1992). ALL of the PCGs in *M. acuminata* mitochondrial genome possess common triplet initiation codons ATN (ATA for *COII*, *ATP8*, *ND1*, ATT for *ND2*, *COI*, *ND3*, *ND5*, and *ND6*, ATG for *ATP6*, *COIII*, *ND4*, *ND4L*, and *Cytb*). All of the PCGs stop with complete termination codons (eleven with TAA and two with TAG).

The *M. acuminata* mitochondrial genome contains 22 tRNA genes ranging from 62 to 71 bp. All the tRNA genes could be folded into a typical cloverleaf secondary structure except for tRNA*^Ser (AGN)^*, due to the deficiency of the dihydrouridine (DHU) arm. The lrRNA was 1314 bp in length with an A + T content of 79.4%, while the srRNA is 786 bp long with an A + T content of 77.5%.

The control region is located between srRNA and tRNA*^Ile^* and is 1080 bp in length with an A + T content of 87.5%, which is the most A + T-rich region of this mitochondrial genome. The A + T content of the whole genome, PCGs, tRNAs, and rRNAs was 75.6%, 73.7%, 75.9%, 78.6%, respectively.

Nineteen coleopteran species (including other 18 species from infraorder Polyphaga in NCBI) were selected to reconstruct a phylogeny with *M. acuminata*. Phylogenetic relationship was inferred from phylogenetic analysis of the 13 protein-coding genes and aligned by mafft v7.427, and 13 ML tree was constructed for each protein by RAxML v8.2.11. Then, the 13 ML trees were combined into one phylogenetic tree by astral v5.7.3. The phylogenetic tree can be visualized by evolview v2 (https://evolgenius.info//evolview-v2/). The phylogenetic tree (Figure 1) revealed *M. acuminata* cluster was more closely related to *Chrysochroa fulgidissima.* Also, the result of phylogenetic tree of 19 species indicated the close relationship between Buprestoidea and Scarabaeoidea at the family level.

**Figure 1.**
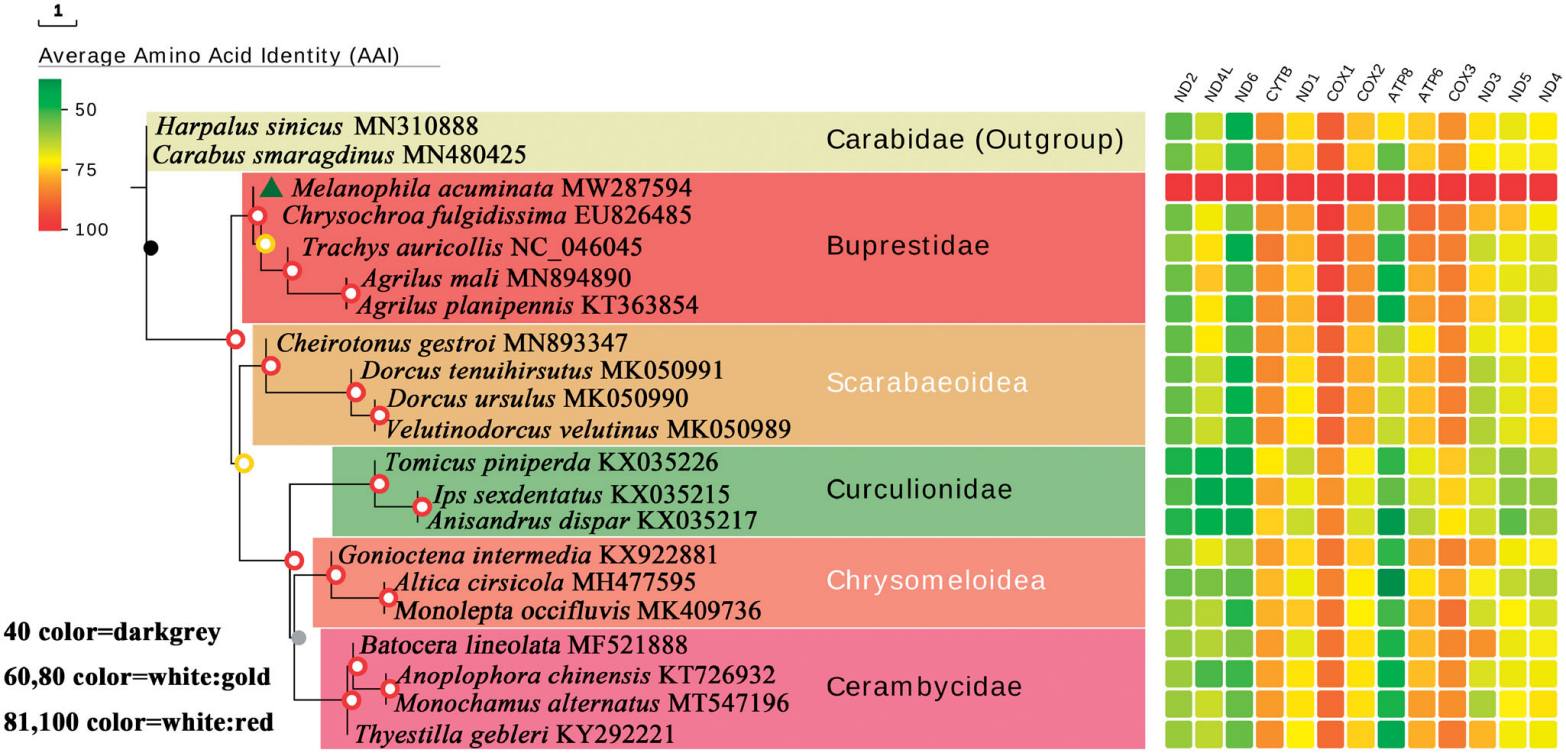
Phylogenetic relationship of the *Melanophila acuminata* and 18 Polyphaga species. Maximum-likelihood phylogeny by using the RAxML v8.2.11 software.

## Data Availability

The genome sequence data that support the findings of this study are openly available in GenBank of NCBI at (https://www.ncbi.nlm.nih.gov/) under the accession no. MW287594. The associated BioProject, SRA, and Bio-Sample numbers are PRJNA670526, SRR12883654, and SAMN16512713, respectively.
